# Infarction Patterns and Recurrent Adverse Cerebrovascular Events in Moyamoya Disease

**DOI:** 10.1155/2022/8255018

**Published:** 2022-03-29

**Authors:** Shao-Chen Yu, Zi-Han Yin, Chao-Fan Zeng, Fa Lin, Long Ma, Yan Zhang, Dong Zhang, Ji-Zong Zhao

**Affiliations:** ^1^Department of Neurosurgery, Beijing Tiantan Hospital, Capital Medical University, Beijing, China; ^2^China National Clinical Research Center for Neurological Diseases, Beijing, China; ^3^Savaid Medical School, University of Chinese Academy of Sciences, Beijing 100049, China; ^4^Center of Stroke, Beijing Institute for Brain Disorders, Beijing, China; ^5^Beijing Key Laboratory of Translational Medicine for Cerebrovascular Disease, Beijing, China

## Abstract

For moyamoya disease (MMD) patients who suffered an acute ischemic attack, the infarction patterns on DWI and its association with recurrent adverse cerebrovascular events (ACEs) after bypass surgery remain unknown. 327 patients who suffered an acute ischemic attack and received following revascularization surgery were retrospectively reviewed and were divided into three patterns according to the lesion number and distribution on DWI that obtained within 7 days of onset: no acute infarction (NAI), single acute infarction (SAI), and multiple acute infarctions (MAIs). We used Cox proportional hazard models to estimate hazard ratios (HR) for associations of infarction patterns and the risk of recurrent ACEs and strokes. Over a median follow-up of 41 months (IQR 26-60), there were 61 ACEs and 27 strokes. Compared to the NAI cohort, patients with SAI (HR, 2.92; 95% CI, 1.41-6.05; *p* = 0.004) and MAIs (HR, 4.44; 95% CI, 2.10-9.41; *p* < 0.001) were associated with higher risk of ACEs recurrences. In analysis adjusted for age and surgery modalities, the corresponding HR was 2.90 (95% CI: 1.41-5.98) for SAI and 4.10 (95% CI: 1.95-8.63) for MAIs, and this effect remained persistent on further adjustment for several potential confounders. Similar but less precise association was found in separate analysis that only takes into account stroke recurrences. Thus, different infarction patterns on DWI imply different risks of recurrent ACEs, and more attention should be paid to prevent ACEs in MMD patients with MAIs.

## 1. Introduction

Moyamoya disease (MMD) is a rare cerebrovascular disorder characterized by bilateral, progressive stenosis in the terminal portions of the internal carotid arteries, with the formation of an abnormal vascular network of basal collaterals. [1] It is a nonnegligible cause of stroke in patients younger than 50 years old, especially in Asia. Majority of MMD patients may manifest as ischemic symptoms, including TIA and cerebral infarctions. [2–4] Without timely and effective treatment, many patients will sooner or later progress into devastating consequences. [5–7] At present, restore perfusion through revascularization surgery remains the most definite therapeutic strategy for preventing future strokes and TIAs, which were adverse cerebrovascular events (ACEs) we concerned most. [8–10] However, there are still many patients experienced recurrent ACEs after revascularization surgery. Hence, discovering simple and feasible imaging parameters that can efficiently define those patients in the early stage is imperative in respect to optimize therapeutic strategy and decrease mortality.

As the most sensitive tool to detect acute cerebral infarctions, diffusion-weighted imaging (DWI) is pivotal in stroke research. [11, 12] Infarction patterns on DWI and their associations with stroke etiologies and long-term prognosis have been well defined. In general stroke patients, it has been testified that, compared to those with no acute infarction (NAI) and single acute infarction (SAI), patients with multiple acute infarctions (MAIs) convey a significantly higher risk of recurrent ACEs. [13, 14] However, it is unclear whether this rule still holds true in MMD patients. So far, rare studies have focused on the infarction patterns in MMD, [15, 16] and none of them studied their underlying prognostic differences.

In this retrospective cohort study, we sought to clarify the infarction patterns and the unequal properties of ACEs recurrences between different infarction patterns among MMD patients who suffered an acute ischemic attack and received revascularization surgery.

## 2. Materials and Methods

### 2.1. Design and Populations

We prepared this manuscript according to the Strengthening the Reporting of Observational Studies in Epidemiology (STROBE) statements.

**Figure 1:**
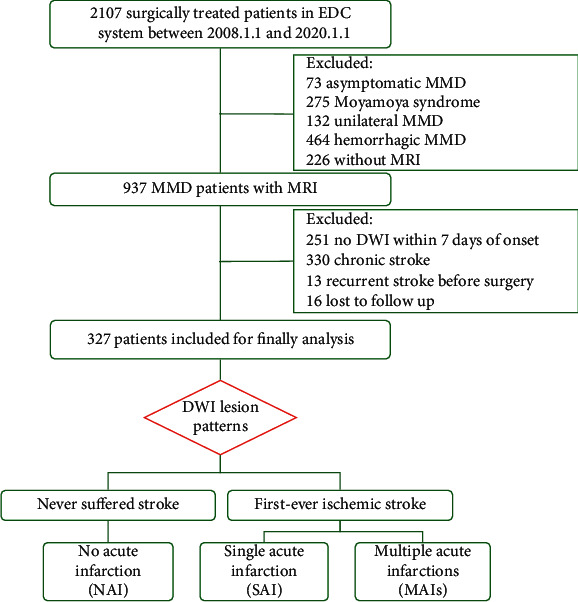
Flow chart for patient enrollment. Abbreviation: EDC: electronic data capture; MMD: moyamoya disease; DWI: diffusion-weighted imaging.

This is a subset of a multicenter retrospective cohort study with prospective follow-up and ongoing online data accrual (electronic data capture system) designed to facilitate longitudinal assessment of patients with moyamoya phenomenon and received revascularization surgery. We screened more than two thousand consecutive patients who underwent revascularization surgery for moyamoya disease at Beijing Tiantan Hospital from January 2008 to January 2020 (Figure 1). Patients that suffered an acute ischemic attack that was confirmed by DWI within 7days of symptom onset were considered as eligible. Asymptomatic patients, patients with hemorrhagic manifestation or with unilateral angiopathy, and those lost to follow-up were excluded. Those combined with other diseases that clearly related to stroke and patients with chronic or recurrent infarctions before surgery were also excluded to achieve higher homogeneity.

### 2.2. Data Collections

Demographic, clinical, and treatment data were extracted from the EDC system. All image data other than DWI were previously provided by the imaging department and were inputted into the EDC system after rechecked by expert neurosurgeons. Specifically, the following data were collected: (1) baseline characteristics (age, sex, comorbidities (hypertension, diabetes mellitus, and hyperlipidemia), and medical history (previous TIA and family history of MMD)), (2) index event characteristics (onset-to-DWI duration and preventive treatment (antiplatelet, anticoagulant, or statin treatment) and presentation), (3) imaging characteristics (perfusion status and cerebrovascular traits (SUZUKI stage, presence of aneurysm, and posterior circulation involvement)), and (4) surgery characteristics (mRS at admission, DWI-to-surgery duration, and surgery modalities). Decrease in regional cerebral blood flow compared to the contralateral hemisphere or cerebellum was considered as perfusion impairment. The Suzuki stages were determined by the anteroposterior and lateral views of the bilateral internal carotid arteries on DSA. If the Suzuki stage on both sides was inconsistent, the higher side shall prevail. Patients with obvious posterior-cerebral-artery stenosis or moyamoya vessel formation on DSA were identified as posterior circulation involvement. Patients who underwent combined revascularization surgery were classified into the direct surgery modality group due to the similarity in surgical procedures and treatment outcomes. Omissions and mistakes were supplemented and corrected by thorough review of the medical records and visual inspection of source brain images as much as possible.

### 2.3. Stroke Patterns Interpretation

All DWI images were centrally assessed by a certified and trained neurosurgeon and a neuroradiologist in random order either through the PACS (picture archiving and communication system) or scanned digital format (patients referred to our center). Discrepant cases were adjudicated by consensus among three or more readers.

Using published templates, [17] patients were grouped into 3 categories (NAI, SAI, and MAIs) according to the lesion number on DWI. Any hyperintense lesions on DWI with corresponding presentation were deemed to be acute infarctions. Uninterrupted lesions visible in contiguous territories were defined as SAI, while MAIs refers to multiple noncontiguous hyperintense lesions that were topographically distinct. SAI were further classified into small perforator artery infarct (diameter < 2 cm), large perforator artery infarct (diameter ≥ 2 cm), pial infarct, large territorial infarct, and border-zone infarct. MAIs were further classified into infarcts in unilateral anterior circulation, infarcts in bilateral anterior circulation, infarcts in posterior circulation, infarcts in both anterior and posterior circulation, and infarcts in multiple border-zone areas, similar as previous studies. [18, 19]

### 2.4. Follow-Up and Outcomes

Discharged patients are advised to visit the clinic regularly, usually at 3 months, 6 months, and every year thereafter. Those did not show up on time are followed up via phone interviews (patients or their next of kin) by participating investigator who was masked to clinical and imaging characteristics. Detailed description of the follow-up events and neurological status were recorded. Follow-up was complete through October 2021. Primary end point was recurrent ACEs with subsidiary analyses focused only on recurrent strokes. We define ACEs as any stroke or TIA of cerebrovascular origin that developed beyond 30 days after surgery. Stroke was defined as a rapid onset of focal retinal or cerebral deficit lasting > 24 hours. TIA was defined as a new retinal or cerebral dysfunction sustained < 24 hours with no new lesion on CT or MRI.

### 2.5. Statistical Analysis

Continuous and categorical variables were presented as median (IQR) and *n* (%), respectively. Kruskal-Wallis test, *χ*^2^ test, or Fisher's exact test were taken to compare differences between different infarction patterns where appropriate. Kaplan-Meier curves with a log-rank test were constructed to describe and examine the recurrences of ACEs over time between different infarction patterns. Hazard ratios (HRs) along with 95% confidence intervals (CIs) for recurrent ACEs were calculated using Cox proportional hazard models and were adjusted for (1) established confounders (age and surgery modalities) (model 1), (2) model 1 plus potential confounders in the present study (age, surgery modalities, and posterior circulation involvement) (model 2), and (3) model 2 plus potential confounders previously reported (age, surgery modalities, posterior circulation involvement, and previous TIA) (model 3), respectively. Subsidiary analyses focused only on recurrent strokes were then conducted to examine the similar association. Additional sensitivity analyses restricted to patients exempted from repeated revascularization were applied to verify the robustness of the results. The Bonferroni method was used to avoid bias caused by multiple comparisons when necessary. Age is included in the COX regression model as a binary variable (≤18 vs >18 years old), and missing data were not imputed. A two-sided *α* < 0 · 05 was considered statistically significant. Statistical analyses were done using R, version 3.6.3 (R Foundation, Vienna, Austria) and SPSS, version 24 (IBM Corporation, Armonk, NY).

## 3. Results

### 3.1. Baseline Characteristics

From January 2008 to January 2020, a total of 2107 patients with moyamoya phenomenon in the EDC system were screened. After stepwise exclusion, 327 patients who suffered an acute ischemic attack and received revascularization surgery were included for final analysis (flowchart for enrollment was shown in Figure 1). Among them, 122 (37.3%) patients had NAI, 126 (38.5%) patients had SAI, and 79 (24.2%) patients had MAIs on DWI.  Table 1 shows the summary of patient characteristics (52% women, median age 35 [range 3–63]), stratified by infarction patterns. Baseline characteristics were well balanced among the three cohorts, except patients with MAIs were more likely to be bilateral perfusion impaired, patients with SAI tended to receive revascularization surgery within 3 months after symptom onset, and patients with NAI were more likely to have a history of TIA but a lower admission mRS.

### 3.2. Infarction Patterns

122 patients did not show any infarction on DWI, and the rest 205 (62.7%) patients showed at least one infarction, consisting of 126 (61.5%) SAI and 79 (38.5%) MAIs. Regarding patients with SAI, large territorial infarction was the most common pattern, observed in 47 patients (37.3%), followed by border-zone infarction in 34 (27.0%), small perforator artery infarction (diameter<2 cm) in 20 (15.9%), pial infarction in 18 (14.3%), and large perforator artery infarction (diameter ≥2 cm) in 7 (5.6%). While for patients with MAIs, infarcts exhibit in unilateral anterior circulation was the most common, observed in 36 patients (45.6%), followed by multiple border-zone in 18 (22.8%), bilateral anterior circulation in 13 (16.5%), anterior and posterior circulation in 11 (13.9%), and only 1 (1.3%) patient developed MAIs in posterior circulation.

### 3.3. Stroke Patterns and Risk of Recurrent ACEs and Strokes

**Figure 2:**
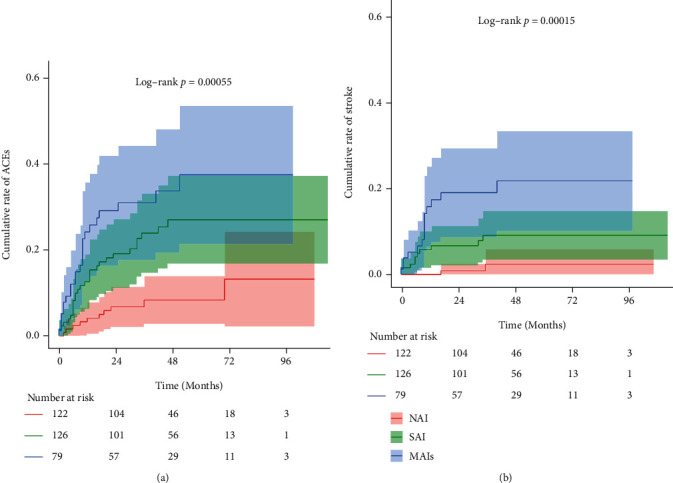
Cumulative Kaplan-Meier curves for recurrent ACEs (a) and strokes (b) during follow-up according to infarction patterns.

Over a median follow-up of 41 months (IQR 26-60, total 14162 months at risk), there were 61 ACEs and 27 strokes, and majority of them were developed in the first 2 years (50/61 for ACEs and 22/27 for stroke). Among those without ACEs, 94% were followed-up for over 2 years. Recurrent ACEs rates per 100 person-year of follow-up across the 3 stroke patterns (NAI, SAI, and MAIs) were 2.3, 6.0, and 8.8, respectively. Kaplan-Meier curves in Figure 2 show cumulative rate of ACEs and stroke among different infarction patterns. Comparing to the NAI cohort, patients with SAI (HR, 2.92; 95% CI, 1.41-6.05; *p* = 0.004) and MAIs (HR, 4.44; 95% CI, 2.10-9.41; *p* < 0.001) were associated with higher risk of ACEs recurrence. In analysis adjusted for previously documented confounders (age and surgery modalities), the HR of recurrent ACEs for patients with MAIs was 4.10 (95% CI: 1.95-8.63, *p* < 0.001). This effect persisted (HR 4.55, 95% CI 2.01-8.96, *p* <0.001) when further adjusted for confounders in the present study (posterior circulation involvement). After additional adjustment for previously reported potential confounder (history of TIA), this association remain unchanged (HR 4.41, 95% CI 2.10-9.32, *p* < 0.001). A similar but less precise association was observed in a separate analysis that only considered stroke recurrence (*n* = 27, including 23 ischemic and 4 hemorrhagic strokes) (Table 2). Sensitivity analyses restricted to patients received single revascularization surgery that yielded consistent results as shown in supplemental materials (available here).

## 4. Discussions

In this research, we clearly depicted the infarction patterns of MMD patients who suffered an acute ischemic attack and found a significant association between infarction patterns and recurrent ACEs after revascularization. This effect was not affected by established or potential clinically relevant confounding factors. Subsidiary analyses revealed a similar but less precise association with recurrent strokes due to the extremely low event of stroke in patients with NAI (*n* = 2). In addition, these positive associations were further confirmed by serial sensitivity analyses.

Due to the rarity of disease and dilemma in rapid access to MRI scan, the infarction patterns of MMD patients remain largely unknown. Here, we found that, among MMD patients who suffered an acute ischemic attack, approximately 60% had positive lesions on DWI, and nearly 40% of them may present as MAIs, which is in the range of previous reported 16%-50% for stroke patients in general. [19–23] Different infarction patterns may occur as a consequence of obstruction of major arteries, thrombosis, or collapse of moyamoya vessels and collateral failure, alone or at the same time. Recently, Dong et al. [15] found that embolism (83.7%) was the most likely mechanism in MMD patients who suffered an acute ischemic stroke, and majority of them showed good collateral status (86%). In our data, infarctions caused by hemodynamic damage alone account for only 15.9% (52/327), suggesting mechanism other than collateral failure should be considered. Besides, Ji et al. [16] indicated that age-specific infarction patterns exist in MMD; on the contrary, we denoted no significant differences in infarction patterns between child-onset (< 18 years old) and adult-onset patients when dichotomized it into SAI and MAIs (*p* = 0.828, data not shown), suggesting a disease-specific infarct mechanism to explain this phenomenon instead of age.

Most importantly, our study demonstrates that among MMD patients who suffered an acute attack, the increase in the number of acute infarctions was positively (*p* value for trend = 0.001) and independently associated with higher risk of recurrent ACEs after revascularization surgery, which is in line with previous studies confine to patients with specific TOAST subtypes. [14] The worldwide TIAregistry.org project, which focused on the one-year risk of major cardiovascular events in patients with TIA or minor stroke (including all the five TOAST subtypes), had shown that patients with MAIs had over two folds higher risk than those with NAI. [24] Apart from this, the subgroup analysis of the CHANCE trial recruited 1089 patients with TIA or minor stroke (excluded patients with cardioembolism and with other determined pathogenesis) and demonstrated that patients with MAIs had nearly 6 times the risk of recurrent stroke compared to those with NAI. Meanwhile, MAIs put the patient at a constant high risk of stroke although it benefited the most from dual antiplatelet treatment [18]. Tomohisa et al. [22] followed up 272 cryptogenic stroke patients for more than 3 years and found that MAIs were independently associated with recurrent stroke and all-cause mortality. In the current study that restricted to MMD, patients with MAIs had hazard ratios for recurrent ACEs and stroke nearly 4 and 10 times more than the hazard ratios in the reference category of NAI. All these along with our findings support that, in spite of disparities in stroke etiology, patients with MAIs share common unstable sources of recurring ACEs.

However, we currently have no credible explanation for such a distinct association. We speculate that brain tissue vulnerable to ischemic attacks are broader in patients with MAIs, and it is unlikely to correct established hypoperfusion with focal revascularization, let alone to reverse the natural history of MMD [25]. In addition to hemodynamic compromise, there are increasing evidence that supports the causality between thromboembolic mechanisms and MMD relevant infarctions [15, 26–28]. Microembolic singles are frequently detected by transcranial Doppler before and after surgery in MMD patients, and their association with previous ischemic events and predictive value of future cerebral events has been verified in several TOAST subtypes [26, 29–31]. Unless back to normal, the extensive moyamoya network may constantly serve as a fountain of emboli thereby contribute to subsequent ACEs. Interestingly, Caplan et al. support the hypothesis that infarction in MMD is resulted from the combination of hypoperfusion and embolism [32]. Taken together, we suggest that mechanism by which MAIs increase the risk of recurrent ACEs is heterogeneous, and the one-size-fits-all strategy (revascularization) alone is insufficient to eliminate the high risk of recurrent ACEs for patients with MAIs. The CARESS trial [33] and the CLAIR trial [34] have shown the superiority of dual antiplatelet therapy in reducing embolic signals. Given the potential causality between embolic singles and higher risk of ACEs in patients with MAIs, we thus wonder whether the combination of revascularization surgery and antithrombotic agents would bring extra benefits to certain MMD patients, such as those with MAIs. However, this is beyond the scope of our study and further researches are warranted to address this conundrum.

Our study highlighted the value of baseline DWI in risk stratification of long-term ACEs recurrences, and we thought our findings may potentially contribute to better and more individualized treatment strategies. Considering that most of the ACEs (82%) and strokes (81.5%) occurred within two years after surgery, we strongly recommend that patients with MMD suffering from acute ischemia, especially those with MAIs, should be followed up regularly within two years after surgery. Further clinical trials are warranted to address whether dual antiplatelet therapy can provide additional benefits in patients with MAIs that show embolic pattern.

As strength, our cohort study demonstrated a strong association between baseline imaging parameter (stroke patterns) and long-term prognosis after surgery through a relatively large sample size. However, our study is subject to certain limitations. First, selection bias is inevitable due to its retrospective nature, although we have tried our best to cover all the eligible patients admitted to one of the largest stroke centers in China. Second, this study only included patients who suffered an acute ischemic attack followed by surgical treatment; therefore, neither the infarct patterns of conservatively treated patients nor the difference in the surgical benefits could be assessed. Third, due to the high prevalence of variations and diversiform changes in the vascular network, the arterial territory in MMD patients may be distinct from others. Thus, detailed infarction patterns determined by normal template may lead to errors. However, these errors were minimized when divided patients into three cohorts according to the number of infarcts. In addition, the length of time from onset to DWI varies; therefore, some of the MAIs may be caused by recurring infarcts during the acute phase, leading to a overestimated ratio of MAIs [35]. Finally, 89.9% (294/327) patients in our study were younger than 50; a full set of vascular examinations were not mandatory; therefore, some of them may harbor other covert source of embolism.

## 5. Conclusion

Overall, our study demonstrated that, for MMD patients who suffered an ischemic attack and received subsequent revascularization surgery, infarction patterns on baseline DWI were significantly associated with the risk of recurrent ACEs thereby could be used as effective tool to define patients remain at high risk of future ACEs. Patients with MAIs should be carefully followed up for at least 2 years, and further studies are warranted to test whether additional antithrombotic agents can provide better curative effect for those patients.

## Figures and Tables

**Table 1:** Baseline characteristics of patients.

Characteristics	Overall (*n* = 327), n (%)	Infarction patterns, n (%)			*p* value
		NAI (*n* = 122)	SAI (*n* = 126)	MAIs (*n* = 79)	
Age, y, (IQR)	35 (14-45)	32 (12-45)	34 (16-44)	39 (24-46)	0.166
≤18	91 (27.8)	40 (32.8)	32 (25.4)	19 (24.1)	0.297
>18	236 (72.2)	82 (67.25)	94 (74.6)	60 (75.9)	
Female	170 (52)	64 (52.5)	64 (50.8)	42 (53.2)	0.939
Current or previous smoking	64 (19.6)	19 (15.6)	35 (23.8)	10 (19.0)	0.26
Alcohol consumption	78 (23.9)	21 (17.2)	35 (27.8)	22 (27.8)	0.094
Comorbidity					
Any	113 (34.6)	39 (32.0)	44 (34.9)	30 (38.0)	0.678
Hypertension	96 (29.4)	33 (27.0)	37 (29.4)	26 (32.9)	0.672
Diabetes	31 (9.5)	6 (4.9)	14 (11.1)	11 (13.9)	0.075
Hyperlipidemia	25 (7.6)	9 (7.4)	12 (9.5)	4 (5.1)	0.50
Family history of MMD	16 (4.9)	4 (3.3)	10 (7.9)	2 (2.5)	0.139
Previous TIA	107 (32.7)	48 (39.3)	36 (28.6)	23 (29.1)	0.143
Time since last TIA> 3 months	42 (12.8)	20 (16.4)	14 (11.1)	8 (10.1)	0.328
mRS at admission					
0-2	307 (93.9)	122 (100)	112 (88.9)	73 (92.4)	0.001
3-5	20 (6.1)	0 (0)	14 (11.1)	6 (7.6)	
Antithrombotic agents	122 (37.3)	37 (30.3)	54 (42.9)	31 (39.2)	0.115
Statin	92 (28.1)	27 (22.1)	39 (31.0)	26 (32.9)	0.169
Onset to DWI duration, d, (IQR)	2 (1-2)	2 (1-2)	2 (1-2)	2 (1-2)	0.851
SUZUKI stage^∗^					
1-2	90 (29.4)	37 (33.3)	26 (21.8)	27 (35.5)	0.094
3-4	155 (50.7)	58 (52.3)	63 (52.9)	34 (44.7)	
5-6	61 (19.9)	16 (14.4)	30 (25.2)	15 (19.7)	
Bilateral CBF decrease †	232 (71.4)	77 (63.1)	94 (75.8)	61 (77.2)	0.037
Present of aneurysms ‡	11 (3.4)	6 (4.9)	3 (2.4)	2 (2.5)	0.484
PCA involvement ‡	107 (32.7)	34 (27.9)	48 (38.1)	25 (31.6)	0.223
Onset to surgery duration, m					
≤ 3	238 (72.8)	95 (77.9)	81 (64.3)	62 (78.5)	0.024
> 3	89 (27.2)	27 (22.1)	45 (35.7)	17 (21.5)	
Surgery modality					
Indirect	195 (59.6)	79 (64.8)	73 (57.9)	43 (54.4)	0.306
Direct	132 (40.4)	43 (35.2)	53 (42.1)	36 (45.6)	
Repeated revascularization	82 (25.1)	28 (34.1)	35 (42.7)	19 (23.2)	0.661
Duration between surgeries, m, (IQR)	8 (6-12)	8 (5-12)	8 (6-11)	9 (6-12)	0.681

^∗^21 patients without digital subtraction angiography before surgery. †2 missing data, 308 patients were assessed via computed tomography perfusion, 5 via arterial spin labeling MR perfusion, 4 via perfusion weighted imaging, and 8 via single-photon emission computerized tomography. ‡310 patients were evaluated by digital subtraction angiography (DSA), and 17 patients were evaluated either by computed tomography angiography (CTA) or magnetic resonance angiography (MRA). Abbreviations: MMD: moyamoya disease; TIA: transient ischemic attack; DWI: diffusion-weighted imaging; CBF: cerebral blood flow; PCA: posterior cerebral artery; IQR: interquartile range.

**Table 2:** Hazard ratios of ACEs and stroke according to infarction patterns.

Stroke patterns	Unadjusted, HR (95% CI)	*p* value	Model 1, HR (95% CI)	*p* value	Model 2, HR (95% CI)	*p* value	Model 3, HR (95% CI)	*p* value
*ACEs*								
NAI	Ref		Ref		Ref		Ref	
SAI	2.92 (1.41-6.05)	0.004	2.90 (1.41-5.98)	0.004	2.81 (1.36-5.80)	0.005	2.93 (1.42-6.07)	0.004
MAIs	4.44 (2.10-9.45)	0.000	4.10 (1.95-8.63)	0.000	4.25 (2.01-8.96)	0.000	4.41 (2.09-9.33)	0.000
*p* value for trend		0.001		0.001		0.001		0.001
*Stroke*								
NAI	Ref		Ref		Ref		Ref	
SAI	4.97 (1.09-22.68)	0.039	4.90 (1.07-22.40)	0.04	4.55 (0.99-20.84)	0.051	4.75 (1.03-21.82)	0.045
MAIs	11.92 (2.71-52.46)	0.001	11.79 (2.67-51.97)	0.001	11.70 (2.65-51.67)	0.001	12.10 (2.73-53.59)	0.001
*p* value for trend		0.002		0.002		0.001		0.001

Model 1, adjusted for age and surgery modality; model 2, model 1 plus posterior circulation involvement; model 3, model 2 plus previous TIA. Abbreviation: CI: confidence interval; ACEs: adverse cerebrovascular events.

## Data Availability

Data supporting the findings of this study are available from the corresponding author upon reasonable request.

## Abbreviations

MMD: Moyamoya disease
ACEs: Adverse cerebrovascular events
TIA: Transient ischemic attack
DWI: Diffusion-weighted imaging
NAI: No acute infarction
SAI: Single acute infarction
MAIs: Multiple acute infarctions
EDC: Electronic data capture
CRESS: Clopidogrel and aspirin for reduction of emboli in symptomatic carotid stenosis
CLAIR: Clopidogrel plus aspirin versus aspirin alone for reducing embolization.

## Supplementary Materials

Supplementary Materials Supplemental materials consist of (1) a table showing detailed infarction patterns and infarction patterns according to surgery modality and age, and (2) a table and a figure showing results of the sensitivity analyses, that are, Hazard Ratios and cumulative Kaplan-Meier curves for recurrent ACEs and strokes during follow-up according to infarction patterns, including patients received single revascularization surgery and (3) an additional checklist of STROBE Statement.
